# Supplementary material for: Dynamic transcriptomic profiles of zebrafish gills in response to zinc supplementation
Source: BMC Genomics. 2010 Oct 11;11:553. doi: 10.1186/1471-2164-11-553 (PMC3091702; doi:10.1186/1471-2164-11-553)
Supplement: Additional file 2 — Interactive Direct Interaction Network representing the molecular interactions between zinc, copper, iron, calcium and proteins encoded by transcripts changed by zinc supplementation. Mini web-site containing index.html and hyperlinked pages in subdirectory describing a Direct Interaction Network automatically generated based on curated interactions contained within the proprietary PathwayArchitect database. Ovals represent proteins and the circles symbolize metal ions. Objects are coloured by their abundance in zebrafish at the time-point they were significantly different from the control is a scale from -4 fold (dark green) to +4 fold (dark red). Where significant differences were found at more than one time-point, the colour overlay shows expression at the first instance. Dark blue squares denote 'binding', and light blue squares 'expression'; green squares stand for 'regulation', green diamonds for 'metabolism', and green circles for 'promoter binding'. Arrow heads indicate directionality of the interaction where annotated. All nodes and edges can be further interrogated by selecting the relative area of the image. [file 1471-2164-11-553-S2.zip › PathwayArchitect Zn xs DIN/713330.html]

# REGULATION:

|  |  |
| --- | --- |
| Type | REGULATION |
| Effect | None |


---

|  |  |
| --- | --- |
| Score | 0 |


---

|  |  |
| --- | --- |
| Reference Count | 5 |


---

|  |  |
| --- | --- |
| Mechanism | Unknown |


---

|  |  |
| --- | --- |
| Reference:0 || Sentence | "We found that while transcriptional induction by zinc can be achieved by elevated zinc concentration alone, induction by cadmium, copper, or H2O2 additionally requires the presence of zinc-saturated metallothionein." |
| PMID | 14612393 |
| Year | 2003 |
| Species | Human |
| Journal | Mol Cell Biol |
| RefScore | 0 |
| Source | PArchNLP |
  |
|  |  |


---

|  |  |
| --- | --- |
 Reference:1 || Sentence | "Zinc acts by inducing intestinal cell metallothionein, which binds copper with high affinity, blocking its absorption, and causing its excretion in the stool." |
| PMID | 2258535 |
| Year | 1990 |
| Species | Human |
| Journal | J Am Coll Nutr |
| RefScore | 0 |
| Source | PArchNLP |
  ||  |  |


---

|  |  |
| --- | --- |
 Reference:2 || Sentence | "In hepatocytes a metallothionein facilitates absorption by a similar mechanism and regulates copper distribution in the liver: incorporation in an apoceruloplasmin, storage and synthesis of copper-dependent enzymes." |
| PMID | 7058150 |
| Year | 1982 |
| Species | Human |
| Journal | Nouv Presse Med |
| RefScore | 3 |
| Source | PArchNLP |
  ||  |  |


---

|  |  |
| --- | --- |
 Reference:3 || Sentence | As a consequence, metallothionein is degraded and the uptake of copper-metallothionein into the lysosomes and the formation of the metallothionein polymer associated copper is reduced. |
| Year | 2004 |
| PMID | 15123354 |
| Species | Rat |
| Journal | J Hepatol |
| RefScore | 1 |
| Source | PArchNLP |
  ||  |  |


---

|  |  |
| --- | --- |
 Reference:4 || Sentence | Many studies have suggested that MT plays a role in the homeostasis of essential metals such as zinc and copper, detoxification of toxic metals such as cadmium, and protection against oxidative stress. |
| PMID | 17018867 |
| Year | 2006 |
| Journal | Exp Biol Med (Maywood) |
| RefScore | 1 |
| Source | PArchNLP |
  |


---

|  |  |
| --- | --- |
